# Establishment and characterization of an immortalized red river hog blood-derived macrophage cell line

**DOI:** 10.3389/fimmu.2024.1465952

**Published:** 2024-09-11

**Authors:** Takato Takenouchi, Kentaro Masujin, Rina Ikeda, Seiki Haraguchi, Shunichi Suzuki, Hirohide Uenishi, Eiji Onda, Takehiro Kokuho

**Affiliations:** ^1^ Institute of Agrobiological Sciences, National Agriculture and Food Research Organization, Tsukuba, Japan; ^2^ Division of Transboundary Animal Disease Research, National Institute of Animal Health, National Agriculture and Food Research Organization, Kodaira, Japan; ^3^ Kyusyu Research Station, National Institute of Animal Health, National Agriculture and Food Research Organization, Kagoshima, Japan; ^4^ Yokohama Zoological Gardens, ZOORASIA, Yokohama, Japan

**Keywords:** African swine fever virus, immortalization, *in vitro* model, macrophages, red river hog

## Abstract

Red river hogs (RRHs) (*Potamochoerus porcus*), a wild species of *Suidae* living in Africa with a major distribution in the Guinean and Congolian forests, are natural reservoirs of African swine fever virus (ASFV) and typically are asymptomatic. Since blood and tissue macrophages of suid animals are target cell lineages of ASFV, RRH-derived macrophages are expected to play an important role in suppressing disease development in infected individuals. In the present study, we successfully isolated RRH-derived blood macrophages using co-culture techniques of RRH blood cells with porcine kidney-derived feeder cells and immortalized them by transferring SV40 large T antigen and porcine telomerase reverse transcriptase genes. The newly established macrophage cell line of the RRH-derived blood cell origin (RZJ/IBM) exhibited an Iba1-, CD172a-, and CD203a-positive typical macrophage-like phenotype and up-regulated the phosphorylation of nuclear factor-κB p65 subunit and p38 mitogen-activated protein kinase in response to the bacterial cell wall components, lipopolysaccharide (LPS) and muramyl dipeptide. In addition, RZJ/IBM cells produced the precursor form of interleukin (IL)-1β and IL-18 upon a stimulation with LPS, leading to the conversion of IL-18, but not IL-1β, into the mature form. Time-lapse live cell imaging with pHrodo dye-conjugated *Escherichia coli* BioParticles demonstrated the phagocytotic activity of RZJ/IBM cells. It is important to note that RZJ/IBM cells are clearly susceptible to ASFV infection and support viral replication *in vitro*. Therefore, the RZJ/IBM cell line provides a unique model for investigating the pathogenesis of ASFV.

## 1 Introduction

Red river hogs (RRHs) (*Potamochoerus porcus*) are the smallest member of the African *Suidae* family. They are natural reservoir hosts of African swine fever virus (ASFV), which is the sole member of the family *Asfarviridae* and the only known DNA arbovirus (1). While ASFV causes a devastating and economically significant disease in domestic pigs, infection with isolates of the same highly virulent ASFV that causes rapid hemorrhagic death in domestic pigs did not lead to clinical disease in RRHs (1). ASFV persists in the sub-Sahara of the African continent via a natural cycle of transmission (the so-called “sylvatic cycle”) between mammalian host species, such as Common warthog (*Phacochoerus africanus*) and RRHs, and soft ticks (*Ornithodorus moubata*) (2).

Macrophages are representative innate immune cells, which are present in all vertebrate tissues and have multiple functions, such as phagocytosis, cytokine production, and antigen presentation (3). They are also recognized as a common target for various viral and bacterial pathogens and contribute to the infection processes of these pathogens (4). Therefore, *in vitro* cultures of macrophages may be useful for the investigation of host-pathogen interactions (5, 6). In this context, RRH macrophages are expected to be an invaluable tool in assessments of the interactions of RRH immune cells with pathogens, such as ASFV.

In the present study, we successfully propagated RRH-derived blood macrophages (RBMs) using a mixed primary culture of peripheral blood cells with porcine primary kidney cells, as described in our previous study (7). We subsequently attempted to immortalize RBMs, established the novel sustainable cell line, RZJ/IBM (RRH, ZOORASIA, Japan/immortalized blood-derived macrophage), and then analyzed the phenotypic characteristics of cells and their susceptibility to ASFV.

## 2 Materials and methods

### 2.1 Ethics statement

A male RRH was properly kept and managed at Yokohama Zoological Gardens, ZOORASIA (Kanagawa, Japan). Heparinized whole blood derived from the RRH was collected when a health check was performed on this animal, and a surplus blood sample was supplied to the present study (approval numbers; #145 by Yokohama Zoological Gardens, ZOORASIA; #0502015 by the National Institute of Animal Health, National Agriculture and Food Research Organization).

Experiments involving lentiviral vectors were approved by the gene recombination experiment safety committee of the Institute of Agrobiological Sciences (#1036465) and the National Institute of Animal Health (#A-20-002), National Agriculture and Food Research Organization.

### 2.2 Isolation of primary macrophages from the peripheral blood of RRH

Macrophage-depleted porcine kidney primary cell cultures, which mostly consist of fibroblastic and myofibroblastic cells, were used as feeder cells for the propagation of RBMs (7). Feeder cells were cultured in growth medium composed of Dulbecco’s modified Eagle’s medium (DMEM) (Nacalai Tesque, Inc., Kyoto, Japan) containing 10% heat-inactivated fetal bovine serum (FUJIFILM Wako Pure Chemical Corp., Osaka, Japan) and supplemented with 25 μM monothioglycerol (FUJIFILM Wako), 10 μg/mL insulin (Sigma, St. Louis, MO), streptomycin-penicillin (100 μg/mL and 100 U/mL, respectively) (Nacalai Tesque), and 5 μg/mL Fungin (InvivoGen, San Diego, CA). Two milliliters of heparinized peripheral blood obtained from a male RRH was directly added to the feeder cell culture in T-150 tissue culture flasks (Sumitomo Bakelite Co., Ltd., Tokyo, Japan) and cultured at 37°C in a humidified atmosphere of 95% air and 5% CO_2_. The culture medium was replaced every 3-4 days. After approximately 10 days, macrophage-like cells that loosely attached to the cell sheet appeared, actively proliferated, and were harvested from the culture supernatant by centrifugation (1,500 r.p.m. for 5 min). As described in previous studies using porcine macrophages (8–11), RBMs adhered to non-tissue culture-grade Petri dishes (NTC-dishes) (Sumitomo Bakelite) and, thus, were separated from other cell types based on this feature. Isolated RBMs were then used in immortalization experiments.

### 2.3 Establishment and subculturing of RZJ/IBM cells

Lentiviral particles carrying the SV40 large T antigen (SV40LT) gene and the porcine telomerase reverse transcriptase (pTERT) gene were prepared as previously described (7). RBMs cultured in 60-mm NTC dishes were exposed to these lentiviral particles in the presence of 6 μg/mL of Polybrene (Nacalai Tesque). The RZJ/IBM cells eventually established were confirmed to be derived from RRHs based on the partial DNA sequence of the mitochondrial 12S ribosomal RNA gene. A species identification analysis was performed at Seibutsugiken Co., Ltd. (Kanagawa, Japan).

In subcultures, RZJ/IBM cells (1×10^6^) were seeded on 90-mm NTC dishes (Sumitomo Bakelite) and continuously passaged every 4-5 days. At each passage, cells were detached using TrypLE express solution (Thermo Fisher Scientific, Waltham, MA), and the number of harvested cells was measured using a Bio-Rad TC20 automated cell counter.

### 2.4 Immunocytochemistry

Cells were seeded on 8-well chamber slides (Asahi Glass Co., Ltd., Tokyo, Japan) at a density of 2×10^5^ cells/well and cultured for 1 or 3 days. After being washed once with DPBS, cells were fixed using 4% paraformaldehyde phosphate buffer solution (Nacalai Tesque), permeabilized with 1% Triton X-100/PBS solution, and blocked with Blocking One Histo (Nacalai Tesque). Cells were then incubated with the primary antibodies at room temperature for 1 h, and the EnVision system (DAKO, Hamburg, Germany) was used to visualize antibody-antigen reactions, according to the manufacturer’s procedure. Cell nuclei were counterstained with Mayer’s hematoxylin solution (FUJIFILM Wako). Stained slides were examined under a microscope (Leica, Bensheim, Germany).

The primary antibodies used for immunocytochemistry were as follows: mouse monoclonal antibodies against CD163 (clone 2A10/11) (Bio-Rad, Hercules, CA), CD169 (clone 3B11/11) (Bio-Rad), CD172a (clone DH59B) (VMRD, Inc., Pullman, WA), CD203a (clone PM18-7) (Bio-Rad), major histocompatibility complex class II (MHC-II) (clone MSA3) (Kingfisher Biotech, Inc., St. Paul, MN), and rabbit polyclonal antibodies against ionized calcium-binding adaptor molecule 1 (Iba1) (FUJIFILM Wako).

### 2.5 Polymerase chain reaction analysis

The successful transduction of the SV40LT and pTERT genes to the RZJ/IBM genome was confirmed by genomic DNA PCR. PCR experiments were performed as described in our previous study (9). The lengths of PCR products derived from the SV40LT and pTERT genes were 128 and 143 base pairs (bp), respectively.

### 2.6 Flow cytometry

RZJ/IBM cells (1×10^6^) were cultured in 90-mm NTC dishes for 3 days, before being treated with or without 1 μg/mL lipopolysaccharide (LPS) (Sigma) for 1 day or 100 ng/mL water-soluble dexamethasone (DEX) (FUJIFILM Wako) for 3 days. Cells were then detached using TrypLE express solution and re-suspended in DPBS (1×10^5^ cells/100 μL) containing mouse monoclonal anti-porcine molecules [CD163 (clone 2A10/11), CD169 (clone 3B11/11), CD203a (clone PM18-7), and MHC-II (clone MSA3)] antibodies. Cells were further labeled with Alexa Fluor 488-conjugated anti-mouse IgG antibodies (Thermo Fisher Scientific), and the number of Alexa Fluor 488-labeled cells and their median fluorescence intensity (MFI) were analyzed using the BD Accuri™ C6 Plus flow cytometer (BD Biosciences). The fluorescence of 40,000 cells was assessed in each experiment. Three independent experiments were performed, and MFI data are expressed as the mean ± standard error of the mean (SEM).

### 2.7 RNA sequencing (RNA-seq) analysis

RZJ/IBM cells (1×10^6^) were cultured in 35-mm NTC dishes for 1 day before being treated with or without 1 μg/mL LPS for 3 h. Total RNA was extracted from cells using the Cytiva RNAspin mini isolation kit (Thermo Fisher Scientific). RNA-seq analyses were performed at Seibutsugiken Co., Ltd. Transcripts per million (TPM) values were used in a quantitative analysis of gene expression levels. Three independent experiments were performed, and TPM data are expressed as the mean ± SEM.

### 2.8 Immunoblotting

RZJ/IBM cells (4×10^5^ cells/well in a 24-well plate) were stimulated with LPS or muramyl dipeptide (MDP) (InvivoGen) in serum-free DMEM at the concentrations indicated. After being incubated at 37°C for 30 min in experiments on the phosphorylation of p38 mitogen-activated protein kinase (MAPK) and the nuclear factor (NF)-κB p65 subunit or for 3 days in experiments on the production of interleukin-1 (IL-1) β and IL-18, culture supernatants were collected and cells were lysed with 200 μL ice-cold lysis buffer [50 mM Tris-HCl (pH 7.4), 150 mM NaCl, 0.5% Triton X-100, and 0.5% sodium deoxycholate] containing cOmplete™ mini protease inhibitor (Roche Diagnostics GmbH, Mannheim, Germany) and PhosSTOP tablets (Roche). Equal volumes of the culture supernatant and cell lysate (25 μL) were separated by sodium dodecyl sulfate-polyacrylamide gel electrophoresis and electro-blotted onto polyvinylidene difluoride membranes (Merck Millipore Ltd., Carrigtwohill, Ireland). The membranes were then incubated with primary antibodies, before being incubated with horseradish peroxidase (HRP)-conjugated secondary antibodies or HRP-conjugated streptavidin. The target proteins were revealed using Chemi-Lumi One ultra (Nacalai Tesque) and detected using a C-DiGit blot scanner (LI-COR, Inc., Lincoln, NE).

The primary antibodies used for immunoblotting were as follows: rabbit monoclonal antibodies against phospho-p38 MAPK (D3F9, Cat. No. #4511), p38 MAPK (D13E1, Cat. No. #8690), and phospho-NF-κB p65 (93H1, Cat. No. #3033) (Cell Signaling Technology, Inc., Danvers, MA); rabbit polyclonal antibodies against NF-κB p65 (Cat. No. #3034, Cell Signaling Technology); biotinylated antibodies against IL-1β (Cat. No. BAF681) and IL-18 (Cat. No. BAF588) (R&D Systems, Inc., Minneapolis, MN); and mouse monoclonal antibodies against GAPDH (MAb 6C5) (Cat. No. 5G4, HyTest Ltd., Turku, Finland).

### 2.9 Phagocytotic assay using pHrodo-labeled *Escherichia coli* BioParticles

RZJ/IBM cells (1×10^6^) were cultured in 35-mm glass-bottomed dishes (Asahi Glass Co., Ltd.) containing growth medium. The next day, 20 μg/mL of pHrodo dye-conjugated *E. coli* BioParticles (Thermo Fisher Scientific) was added, and cells were subjected to time-lapse recording at 37°C for 5 h using an inverted fluorescence microscope (Olympus IX-81, Tokyo, Japan). The mean intensity of fluorescence emitted by pHrodo was quantified by analyzing captured photographs using the software MetaMorph, version 7.6 (Molecular Devices, Downingtown, PA). Three independent experiments were performed, and data are expressed as the mean ± SEM.

### 2.10 ASFV titration

The ASFV field isolates, Armenia07, Kenya05/Tk-1, and Espana75, were kindly provided by Professor Sanchez-Vizcaino (Universidad Complutense de Madrid, Spain). These isolates were routinely maintained in primary porcine alveolar macrophage (PAM) cell cultures and stored in aliquots at -80°C until used. PAM cells were prepared from 8-week old Landrace-Large White-Duroc crossbred pigs using the broncho-alveolar lavage procedure as previously described (12), and were then cultured in RPMI1640 (Nacalai Tesque) supplemented with 10% FBS and antibiotics. The Lisbon60 isolate was kindly provided by Dr. Genovesi (Plum Island Animal Disease Center, USA) and serially passaged in Vero cell cultures to establish Vero cell-adapted Lisbon60V viruses.

Virus titrations for ASFV isolates were analyzed using cytopathic effects (CPE) and hemadsorption (HAD) assays, as previously described (6). Briefly, RZJ/IBM cells and immortalized porcine kidney-derived macrophage (IPKM) cells established in our previous study (7) were seeded on 96-well cell culture plates, and 100 µL of ten-fold serially diluted samples were inoculated into the wells. The presence of CPE was detected by the disruption of monolayer cell sheets caused by viral infection and examined by microscopy at 2 or 3 days post-inoculation (dpi). HAD assays were performed using porcine red blood cells, and rosette formation was also examined by microscopy at 2 or 3 dpi.

### 2.11 ASFV growth assay

To evaluate ASFV production, RZJ/IBM and IPKM cells (2×10^6^) were seeded in T-25 tissue culture flasks (Sumitomo Bakelite Co., Ltd.) and inoculated with ASFV isolates at a multiplicity of infection (MOI) of 0.01. After cells had been incubated at 37°C for 1 h, the inoculum was removed, cells were washed three times with DPBS, and growth medium was added. Culture supernatants were collected at 0, 1, 2, 3, 4, and 5 dpi, and viral titers against IPKM cell cultures were examined based on CPE, as described in a previous study (6). Viral titers are expressed as TCID_50_/mL (50% tissue culture infectious dose per mL). All experiments with ASFV were performed at the Biosafety Level 3 facility of the National Institute of Animal Health and were approved by the Japanese national authority (Permit No. 32).

Statistical analyses were conducted using KaleidaGraph software (Synergy Software, Reading, PA, USA). The Student’s *t*-test was used for paired data, and differences with p-values <0.05 were considered to be significant.

## 3 Results

### 3.1 Expansion and isolation of RBMs

**Figure 1:**
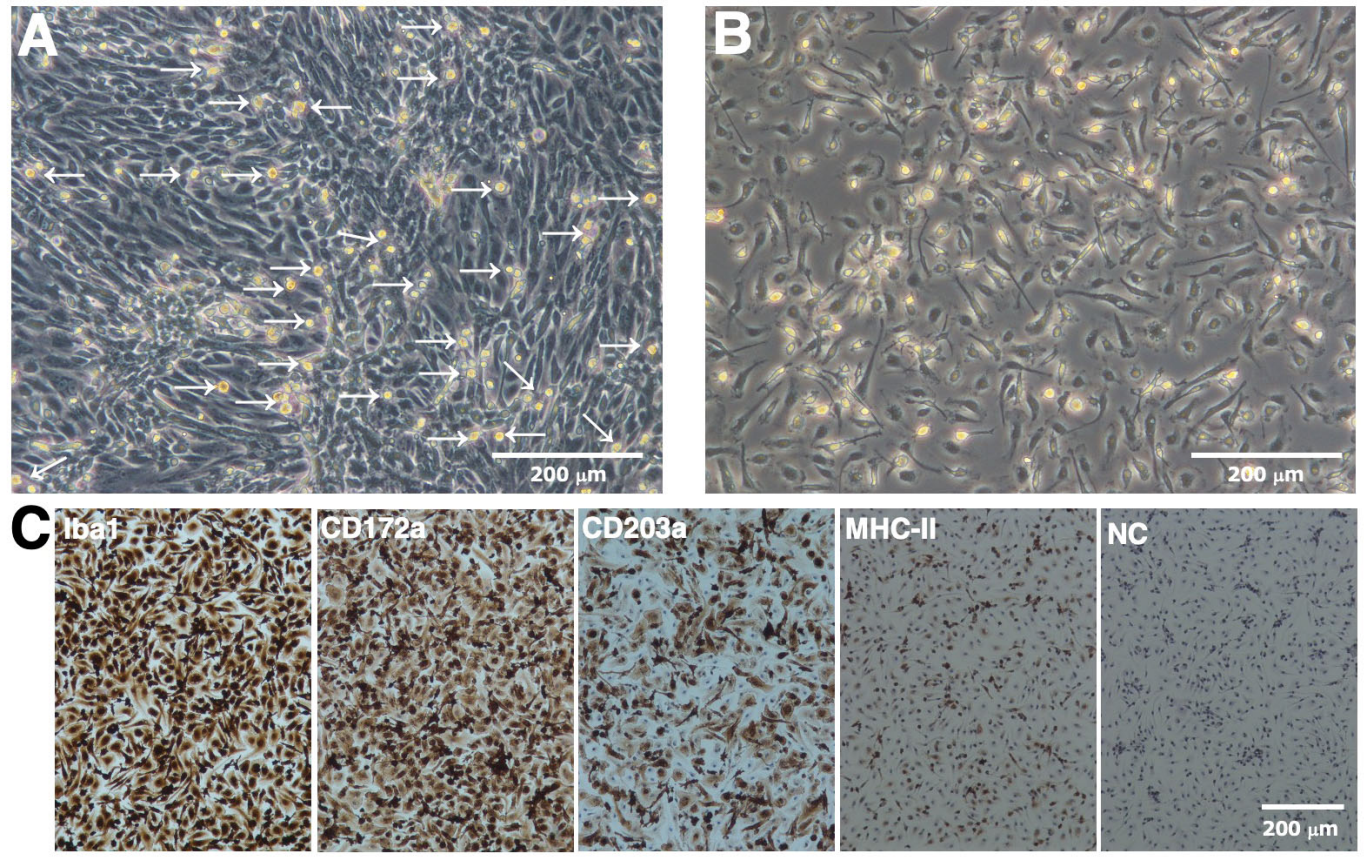
Proliferation and isolation of primary RBMs. RBMs proliferated under the mixed culture conditions of RRH whole blood with primary porcine kidney feeder cells **(A**, arrows). The macrophage-like morphology of isolated RBMs was observed under a phase-contrast microscope **(B)**. RBMs were seeded on 8-well chamber slides and cultured for 1 day. Cells were then fixed using 4% paraformaldehyde phosphate buffer solution and immunostained with specific antibodies against cell markers of macrophages (*brown*) **(C)**. No specific staining was observed when cells were treated without primary antibodies (NC: negative control in C). All nuclei were counterstained with hematoxylin (*blue*) **(C)**. Images are representative of two independent experiments.

RBMs proliferated under the mixed culture conditions of RRH whole blood with primary porcine kidney feeder cells (**Figure 1A**). RBMs loosely attached to the monolayer of feeder cells at the bottom of tissue culture flasks; therefore, they were easily released from the feeder layer by pipetting, harvested from the culture supernatant by centrifugation, and isolated from other types of cells based on their ability to adhere to NTC dishes (**Figure 1B**). Immunostaining results showed that the majority of isolated RBMs were positive for macrophage markers (Iba-1 and CD172a) (**Figure 1C**). The number of CD203a-positive cells, another marker of macrophages, was slightly decreased compared to that of Iba-1 or CD172a-positive cells (**Figure 1C**). Some populations of RBMs were also positive for the differentiated macrophage-specific marker, MHC-II (**Figure 1C**).

### 3.2 Establishment and characterization of RZJ/IBM cells

**Figure 2:**
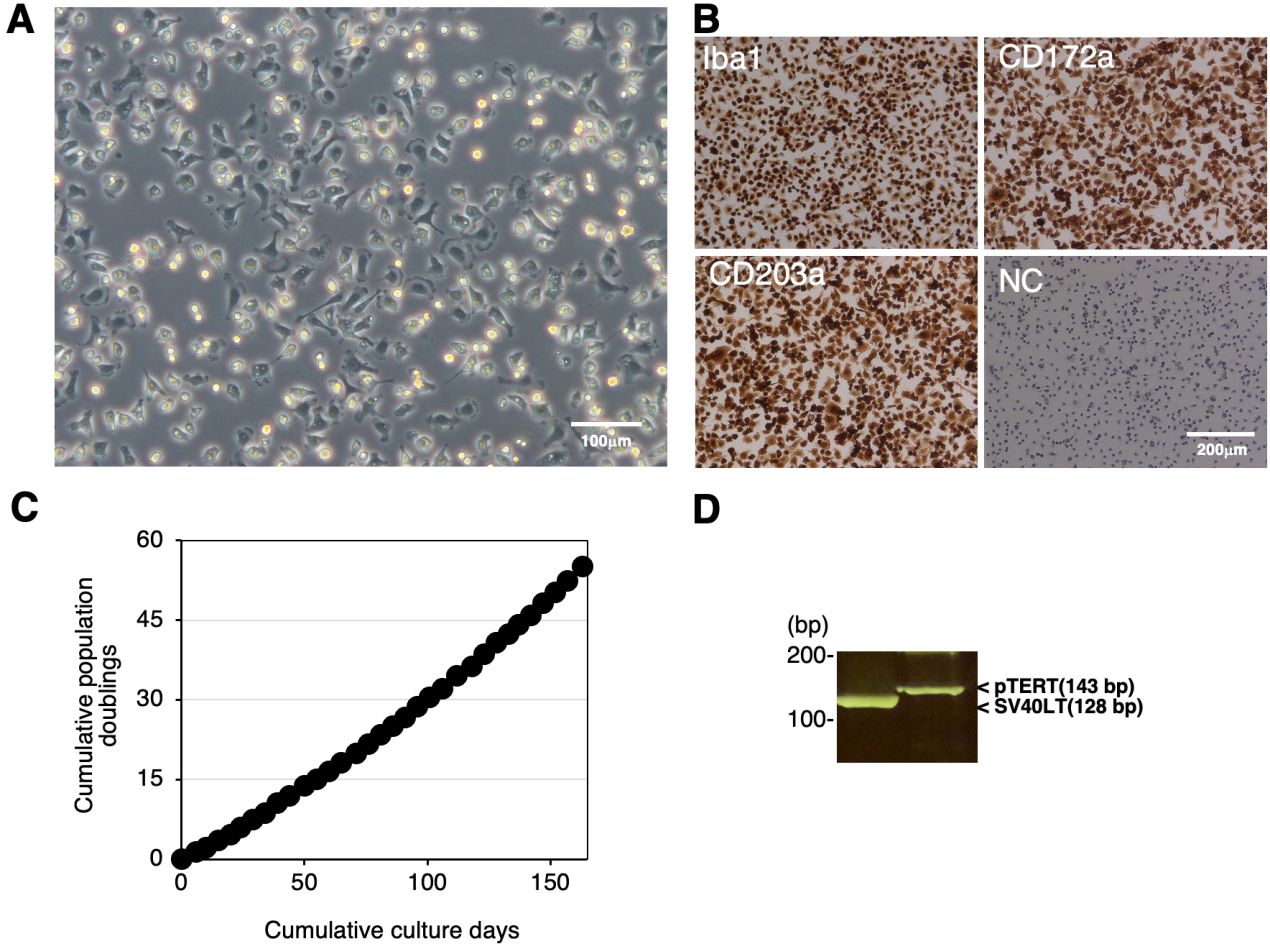
Establishment and characterization of RZJ/IBM cells. The morphology of RZJ/IBM cells was observed under a phase-contrast microscope **(A)**. They were seeded on 8-well chamber slides and cultured for 1 day. Cells were then fixed using 4% paraformaldehyde phosphate buffer solution and immunostained with specific antibodies against the cell markers of macrophages (*brown*) **(B)**. No specific staining was observed when cells were treated without primary antibodies (NC: negative control in B). The cumulative population doublings of RZJ/IBM cells were plotted against the duration of the culture period (in days) **(C)**. The PCR products generated from the SV40LT (128 bp) and pTERT (143 bp) genes were detected by a genomic DNA PCR analysis **(D)**.

We immortalized RBMs by transfecting both the SV40LT and pTERT genes using lentiviral vectors and established the continuingly proliferating cell line, RZJ/IBM. This cell line exhibited a typical macrophage-like morphology with ruffled membranes and cell processes (**Figure 2A**). Similar to primary RBMs, RZJ/IBM cells were positive for macrophage markers (Iba-1, CD172a, and CD203a) (**Figure 2B**). They stably grew to at least 55 population doublings within 163 days (**Figure 2C**). The transduction of immortalizing genes was confirmed by a genomic DNA PCR analysis (**Figure 2D**).

### 3.3 Enhanced expression of CD169 and MHC-II in RZJ/IBM cells treated with LPS

**Figure 3:**
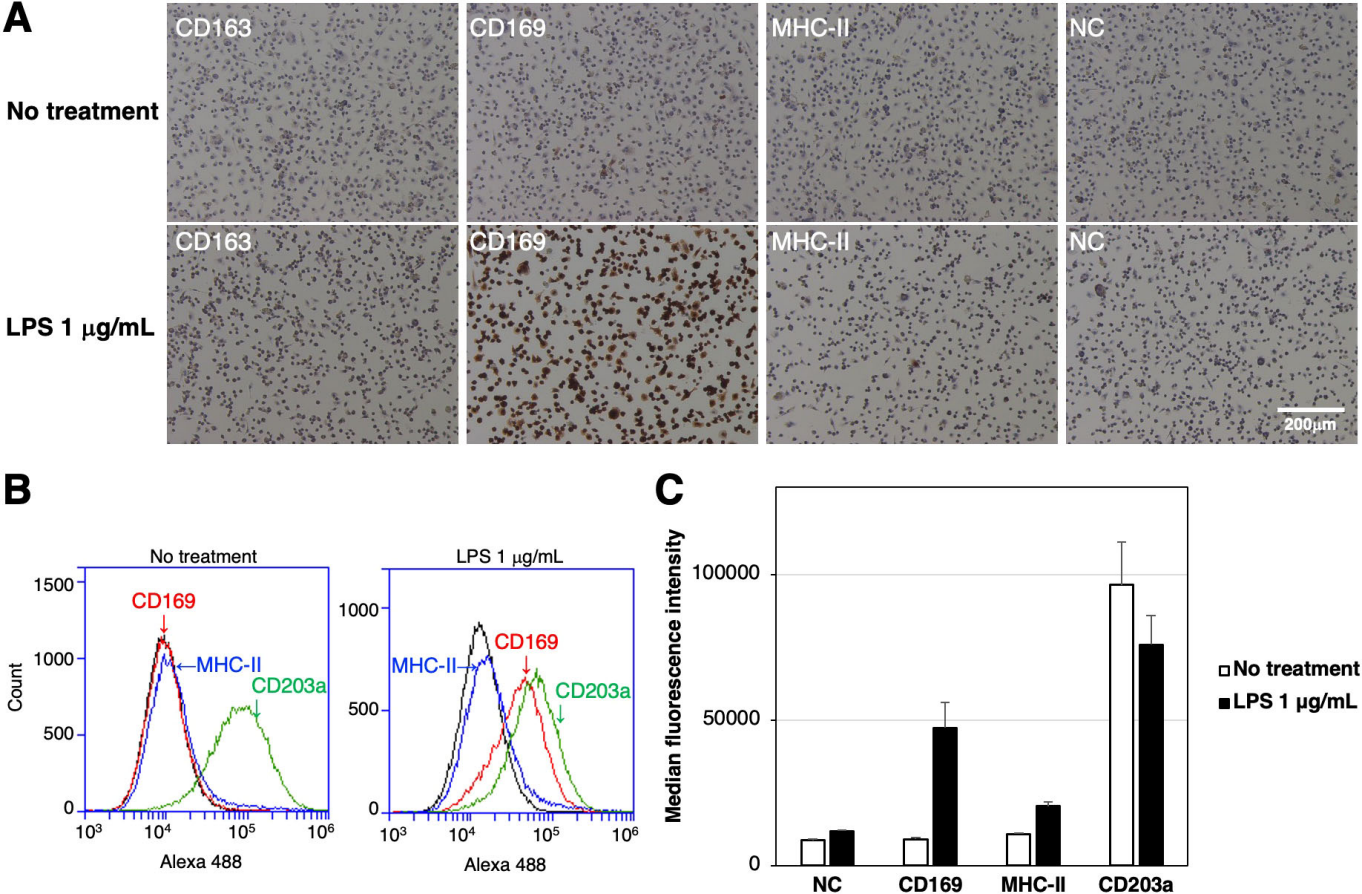
Enhanced expression of CD169 and MHC-II in LPS-treated RZJ/IBM cells. RZJ/IBM cells were treated with (lower panels) or without 1 μg/mL LPS (upper panels) for 1 day, and then immunostained with specific antibodies against cell markers of macrophages (*brown*) **(A)**. All nuclei were counterstained with hematoxylin (*blue*) **(A)**. Cells were also reacted with mouse monoclonal anti-MHC-II (blue line), anti-CD203a (green line), or anti-CD169 (red line) antibodies before being labeled with Alexa Fluor 488-conjugated anti-mouse IgG antibodies (Alexa 488) **(B)**. Cells treated with Alexa 488 alone were used as a negative control (NC) (black line) **(B, C)**. Flow cytometry histograms are representative of three independent experiments **(B)**, and MFI data are expressed as mean ± SEM values **(C)**.

The expression of CD163, CD169, and the differentiated macrophage-specific marker, MHC-II, in RZJ/IBM cells with or without the LPS stimulation was examined by immunostaining. The expression of CD169 was markedly up-regulated in cells treated with LPS (**Figure 3A**). This result was supported by the flow cytometric analysis (**Figure 3B**). The quantitative analysis revealed that the MFI values of CD169-positive and MHC-II-positive cell populations were increased by 5.3- and 1.9-fold, respectively, with LPS treatment compared to untreated group (**Figure 3C**). In contrast, the MFI value of the CD203a-positive cell population was slightly lower in LPS-treated cells than in untreated cells (**Figure 3C**).

### 3.4 Enhanced expression of CD163 in RZJ/IBM cells treated with DEX

**Figure 4:**
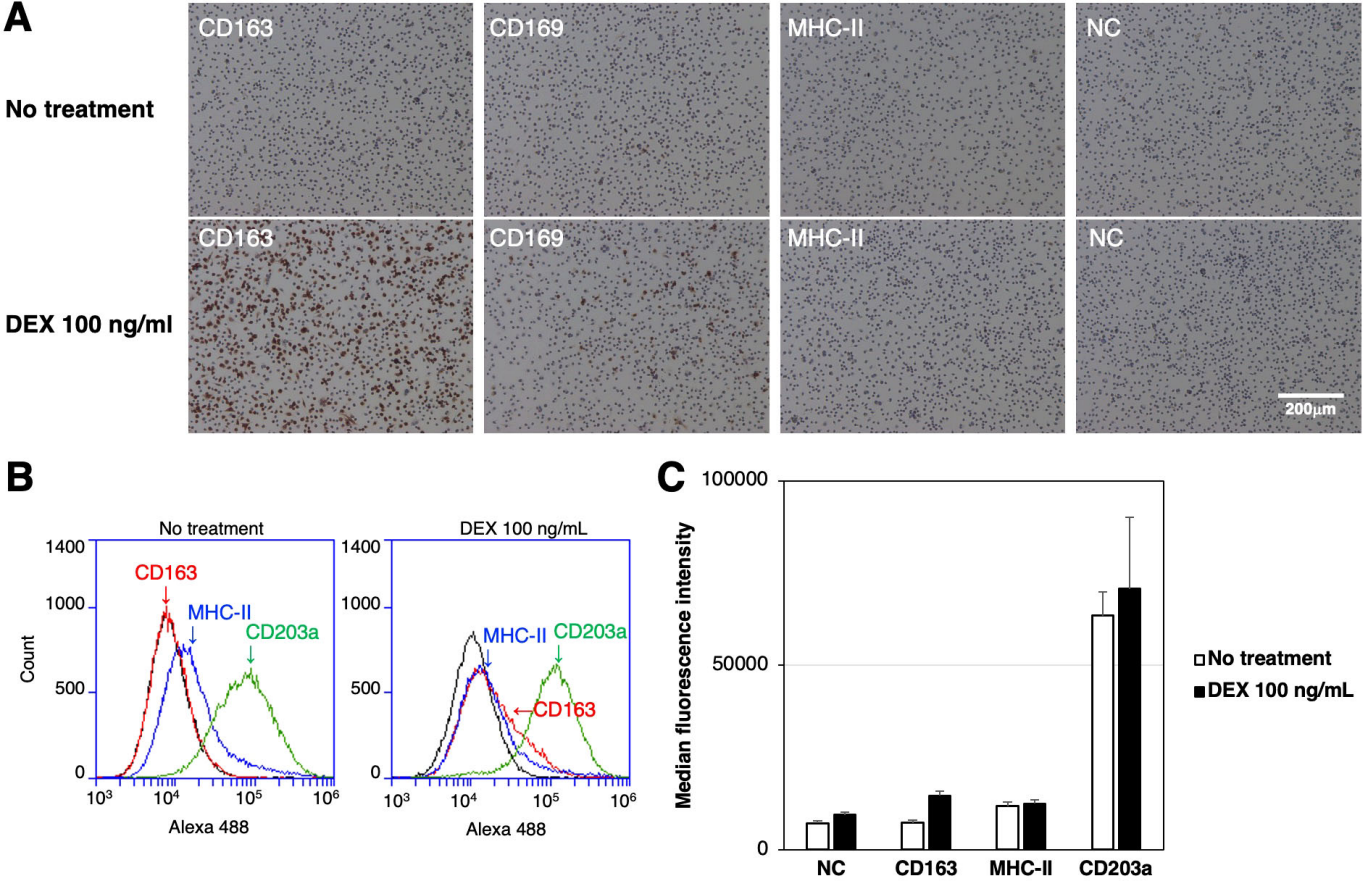
Up-regulated expression of CD163 in DEX-treated RZJ/IBM cells. RZJ/IBM cells were treated with (lower panels) or without 100 ng/mL DEX (upper panels) for 3 days, and then immunostained with specific antibodies against cell markers of macrophages (*brown*) **(A)**. All nuclei were counterstained with hematoxylin (*blue*) **(A)**. Cells were also reacted with mouse monoclonal anti-MHC-II (blue line), anti-CD203a (green line), or anti-CD163 (red line) antibodies before being labeled with Alexa Fluor 488-conjugated anti-mouse IgG antibodies (Alexa 488) **(B)**. Cells treated with Alexa 488 alone were used as a negative control (NC) (black line) **(B, C)**. Flow cytometry histograms are representative of three independent experiments **(B)**, and MFI data are expressed as mean ± SEM values **(C)**.

The expression of CD163, CD169, and MHC-II in RZJ/IBM cells treated with or without the anti-inflammatory steroid, DEX was examined by immunostaining. The up-regulated expression of CD163 was observed in DEX-treated RZJ/IBM cells (**Figure 4A**), and this was supported by quantitative flow cytometry (**Figures 4B, C**). The MFI values of MHC-II-positive and CD203a-positive cells remained unchanged following the DEX treatment (**Figure 4C**).

### 3.5 Induction of pro-inflammatory cytokine and interferon β mRNAs in RZJ/IBM cells upon the LPS stimulation

**Figure 5:**
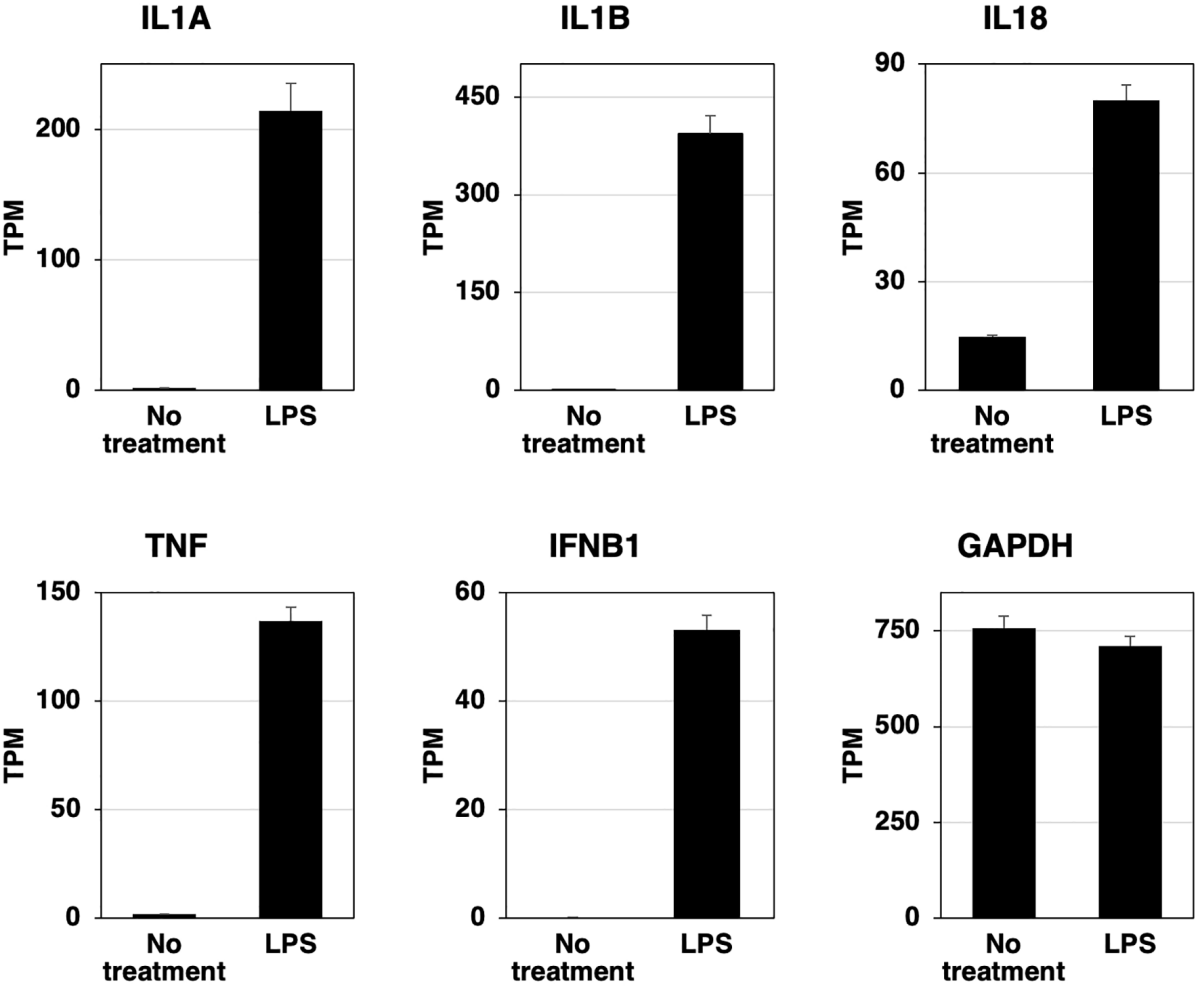
LPS-induced increases in the expression of inflammatory cytokine and interferon β mRNAs in RZJ/IBM cells. Total RNA was recovered from RZJ/IBM cells treated with or without 1 μg/mL LPS, and RNA-seq experiments were performed independently three times. The transcripts per million (TPM) values of the genes indicated were expressed as mean ± SEM values. The TPM value of GAPDH was used as an internal control.

RZJ/IBM cells were stimulated with LPS for 3 h, total RNAs were extracted, and the RNA-seq analysis was performed. The expression of IL-1 family cytokine mRNAs (IL1A, IL1B, and IL18) was markedly up-regulated after the stimulation with LPS (**Figure 5**). The mRNA expression of the pro-inflammatory cytokine, tumor necrosis factor-α (TNF), and interferon-β (IFNB1) was also up-regulated following the stimulation with LPS (**Figure 5**). The expression level of GAPDH mRNA remained stable with or without the LPS stimulation (**Figure 5**).

### 3.6 Phosphorylation of p65 NFκB and p38 MAPK, and the production of IL-1β and IL-18 in RZJ/IBM cells upon the LPS stimulation

**Figure 6:**
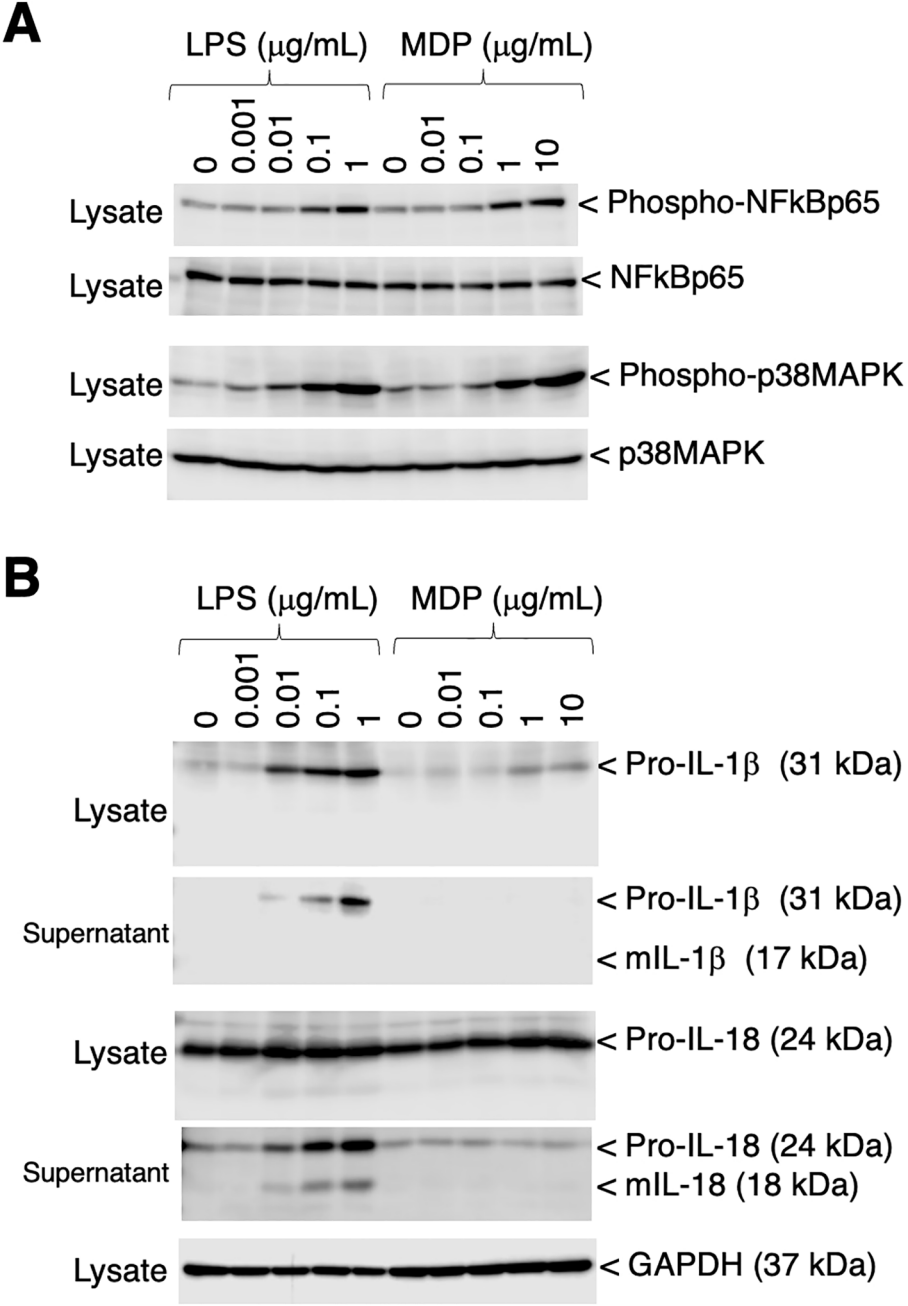
Phosphorylation of p65 NF-κB and p38 MAPK, and the production of IL-1β and IL-18 in RZJ/IBM cells in response to LPS or MDP. The treatment with LPS or MDP induced the phosphorylation of NF-κB p65 and p38 MAPK in a dose-dependent manner (**A**, *first and third panels*). The equivalent protein loading of these molecules was confirmed by immunoblotting with anti-NF-κB p65 or anti-p38 MAPK antibodies (**A**, *second and fourth panels*). The dose-dependent production of pro-IL-1β and pro-IL-18 was also detected in cell lysates (**B**, *first and third panels*) or culture supernatants (**B**, *second and fourth panels*) from RZJ/IBM cells that had been stimulated with LPS for 3 days. The secretion of mIL-18 from LPS-treated RZJ/IBM cells into the culture supernatant was also detected (**B**, *fourth panel*), whereas that of mIL-1β was not (**B**, *second panel*). MDP exerted a negligible effect on the production of pro-IL-1β (**B**, *first panel*) and pro-IL-18 (**B**, *third panel*). GAPDH was used as an internal control (**B**, *fifth panel*). Blots are representative of three independent experiments.

To assess the phosphorylation of p65 NFκB and p38 MAPK, RZJ/IBM cells were stimulated with the bacterial cell wall components, LPS and MDP. Both stimuli induced the dose-dependent phosphorylation of p65 NFκB and p38 MAPK (**Figure 6A**). The production and secretion of IL-1β and IL-18, which are potent pro-inflammatory cytokines, were then investigated. Upon stimulation, these two cytokines are initially produced as inactive precursor forms (31 kDa of IL-1β and 24 kDa of IL-18), followed by the production and secretion of processed active mature forms (17 kDa of IL-1β and 18 kDa of IL-18). The LPS stimulation induced the production of the precursor form of IL-1β (pro-IL-1β), whereas the MDP-induced production of pro-IL-1β was negligible (**Figure 6B**, first panel). Moreover, LPS induced the secretion of pro-IL-1β into the culture supernatant, whereas MDP did not (**Figure 6B**, second panel). LPS also slightly enhanced the production of the precursor form of IL-18 (pro-IL-18) (**Figure 6B**, third panel). Furthermore, a dose-dependent increase in the release of pro-IL-18 and its mature form (mIL-18) into the culture supernatant was observed in LPS-treated RZJ/IBM cells, but not in those treated with MDP (**Figure 6B**, fourth panel).

### 3.7 Phagocytotic activity of RZJ/IBM cells

**Figure 7:**
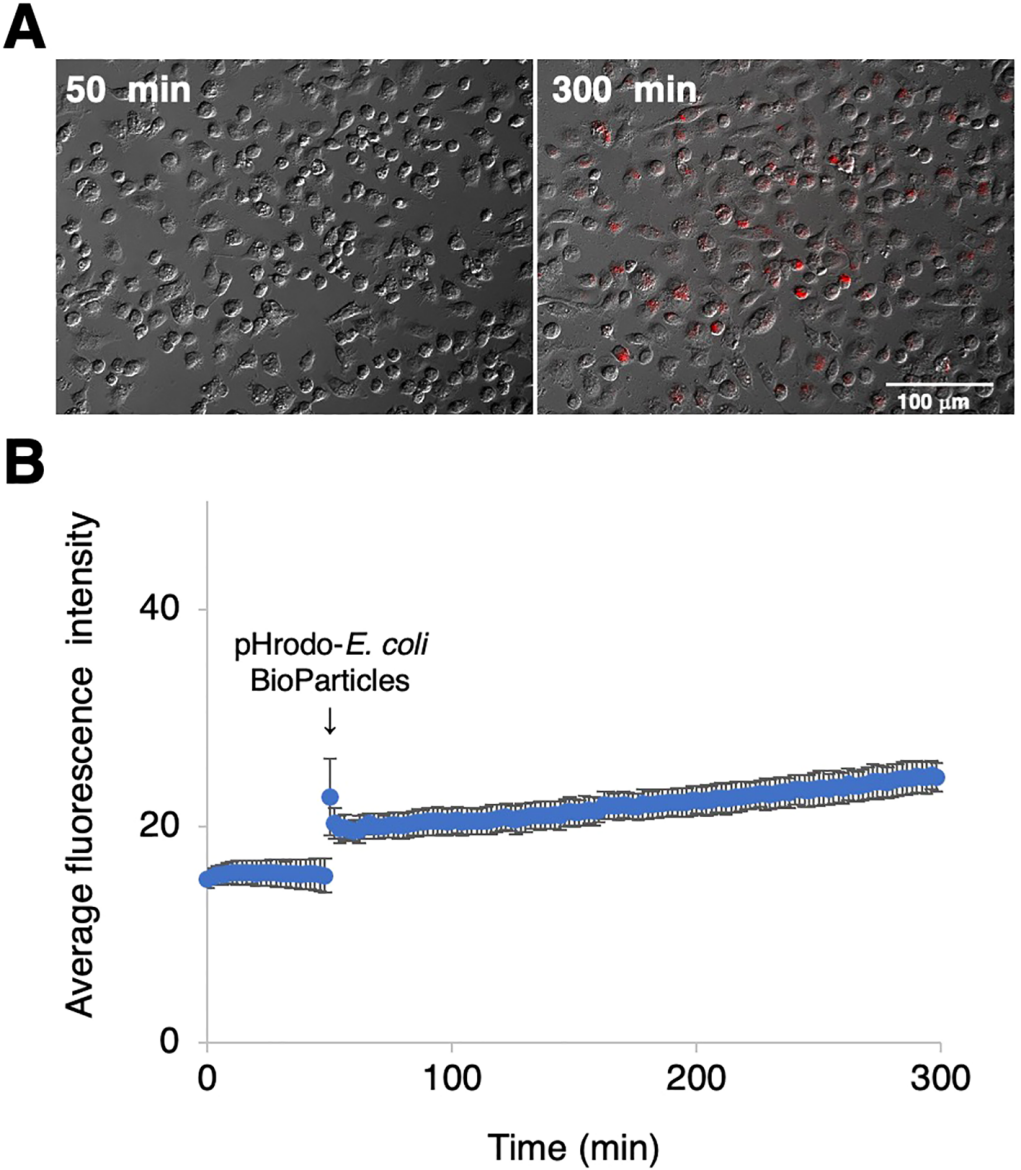
Phagocytotic activity of RZJ/IBM cells. RZJ/IBM cells were treated with pHrodo-labeled *E. coli* BioParticles and monitored via the time-lapse fluorescence imaging of live cells **(A)**. The mean intensity of fluorescence emitted by pHrodo was plotted against the duration of the culture period (in minutes) **(B)**. The experiments were performed independently three times and data are expressed as mean ± SEM values **(B)**.

To examine the phagocytotic activity of RZJ/IBM cells, they were treated with pHrodo-labeled *E. coli* BioParticles and monitored by time-lapse fluorescence imaging. Cells exhibited pHrodo-derived fluorescence representing phagosome maturation after a 4-h incubation (**Figure 7A**). The average intensity of fluorescence slightly increased in a time-dependent manner (**Figure 7B**).

### 3.8 CPE and rosette formation in RZJ/IBM cells upon the ASFV inoculation

**Figure 8:**
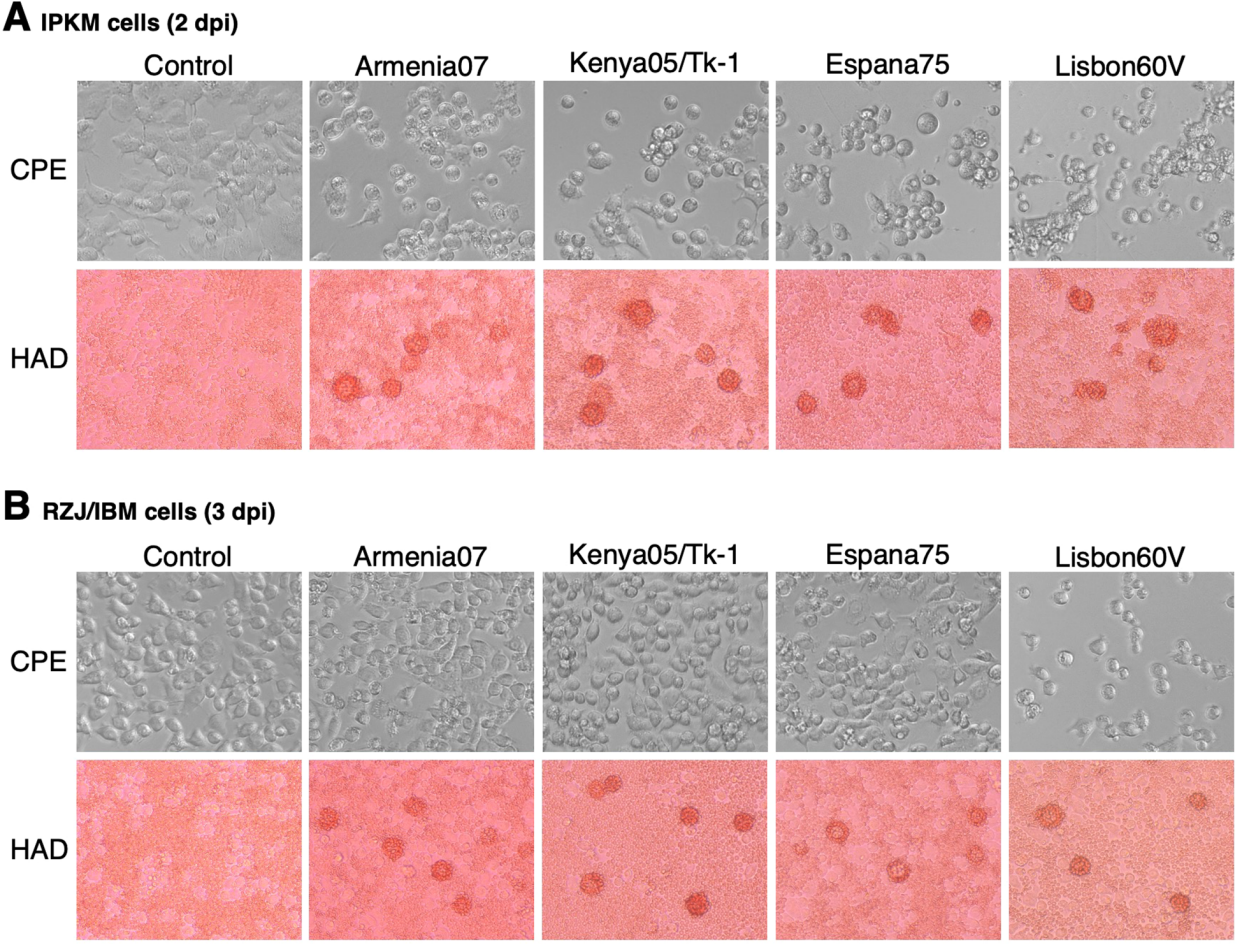
CPE and HAD assays of ASFV-inoculated RZJ/IBM and IPKM cells. The presence of CPE was detected by the disruption of monolayer cell sheets caused by viral infection and examined by microscopy. HAD assays were performed using porcine red blood cells, and rosette formation was examined by microscopy. CPE and rosette formation were detected in IPKM cells inoculated with the virulent ASFV field isolates Armenia07, Kenya05/Tk-1, Espana75, and Vero cell-adapted isolate Lisbon60V (MOI = 0.1) at 2 dpi **(A)**. Rosette formation, but not CPE, was observed in RZJ/IBM cells inoculated with Armenia07, Kenya05/Tk-1, and Espana75 (MOI = 0.1) at 3 dpi **(B)**. CPE and rosette formation were both detected in Lisbon60V-inoculated RZJ/IBM cells (MOI = 0.1) at 3 dpi relative to the findings obtained with mock-infected control cells **(B)**.

The susceptibility of RZJ/IBM cells to ASFV infection was compared with that of IPKM cells, which were used as a positive control. In IPKM cells, CPE and rosette formation in the HAD reaction were both observed following the inoculation with the virulent ASFV field isolates, Armenia07, Kenya/Tk-1, Espana75, and Vero cell-adapted isolate Lisbon60V (**Figure 8A**). Armenia07, Kenya/Tk-1, and Espana75-inoculated RZJ/IBM cells only showed rosette formation without any evidence of CPE (**Figure 8B**). In contrast, RZJ/IBM cells inoculated with Lisbon60V only displayed both CPE and rosette formation (**Figure 8B**).

### 3.9 Propagation of ASFV isolates in RZJ/IBM cells

**Figure 9:**
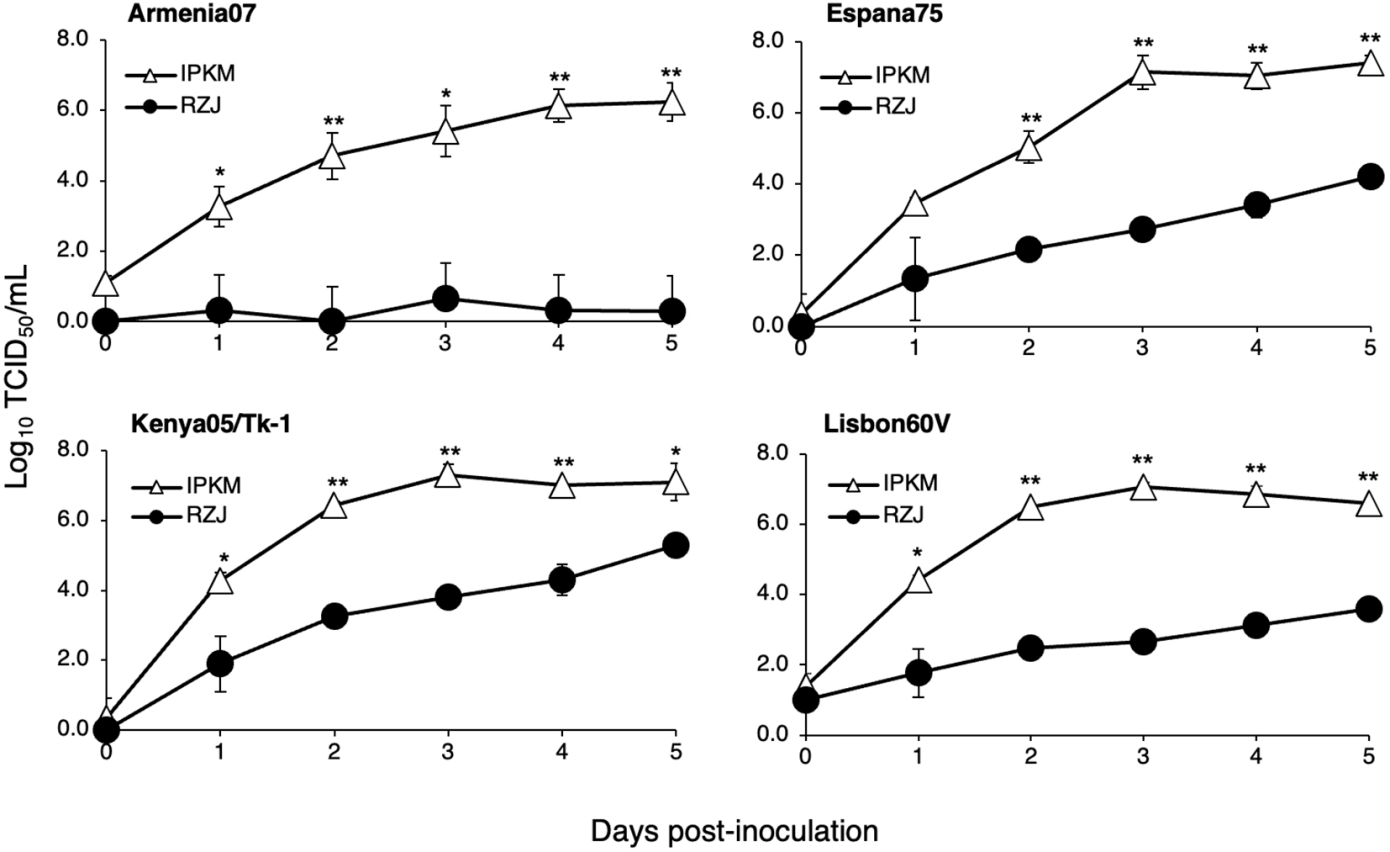
Comparison of ASFV production in RZJ/IBM and IPKM cell cultures. Cell cultures were infected with Armina07, Kenya05/Tk-1, Espana75, or Lisbon60V isolates (MOI = 0.01). Culture supernatant samples were collected at the indicated timepoints. Viral production in the RZJ/IBM (*closed circles*) and IPKM (*open triangles*) cell cultures was estimated by titration experiments with IPKM cells. Data represent the mean and standard deviation of three experiments. Asterisks indicate significant differences in viral production between the RZJ/IBM and IPKM cell cultures (**p < 0.001, *p < 0.05).

We investigated whether RZJ/IBM cells supported the replication of ASFVs. As shown in **Figure 9**, all ASFV strains examined in the present study, namely, Armenia07, Kenya05/Tk-1, Espana75, and Lisbon60V, efficiently propagated in IPKM cell cultures, reaching maximum titers of 10^6^ to 10^7^ TCID_50_/mL at 5 dpi. However, Armenia07 failed to propagate in RZJ/IBM cell cultures under the same culture conditions. Moreover, while other ASFV strains (Kenya05/Tk-1, Espana75, and Lisbon60V) grew in RZJ/IBM cell cultures, their propagation was far less vigorous, with maximum titers reaching only 10^3^ to 10^4^ TCID_50_/mL at 5 dpi.

## 4 Discussion

The present study first reports the establishment of a sustainable cell line of a RRH-macrophage origin using the mixed cell culture technique with porcine primary kidney cells as feeder cells. Given the successful propagation of RBMs, it is clear that the primary kidney cells of the *Sus scrofa* origin support the growth of RRH-derived cells in mixed cultures even though these cells originated from distinct species of different genera. Akin to primary macrophages of other mammalian origins, RBMs adhere to NTC dishes and are easily separated from other cell types. We then immortalized RBMs by introducing both the SV40LT and pTERT genes to acquire the novel cell line, RZJ/IBM.

We previously reported that the LPS stimulation markedly up-regulated the expression of MHC-II in immortalized porcine macrophages (9). The moderate augmentation of MHC-II expression by LPS was observed in RZJ/IBM cells. Furthermore, we noted that CD169 expression was significantly up-regulated in RZJ/IBM cells treated with LPS. CD169, known as sialoadhesin or sialic acid-binding immunoglobulin-like lectin-1 (Siglec-1), is exclusively expressed by specified macrophage populations (13). Since CD169 plays a critical role in cell-to-cell adhesion and cell-pathogen interactions (13), the use of RZJ/IBM allows us to investigate the biological functions of CD169 in RRHs at the molecular and cellular levels.

Regarding the inflammatory response of RZJ/IBM cells, the treatment with LPS induced the phosphorylation of the NFκB p65 subunit and this was followed by the up-regulated expression of various inflammatory cytokine and interferon mRNAs. The LPS-induced production of IL-1β and IL-18 was confirmed at the protein level in RZJ/IBM cells. These results suggest that RZJ/IBM cells exhibit M1 macrophage polarization in the basal state (14). In addition, a DEX-induced increase in CD163 expression was observed in RZJ/IBM cells, which is consistent with our previous findings on IPKM cells (15). CD163 is a scavenger receptor for a hemoglobin-haptoglobin complex and is known as a phenotypic marker of anti-inflammatory M2 macrophages (16). Therefore, as an *in vitro* model, M1/M2 polarity in RZJ/IBM cells may be reproduced by adjusting culture conditions. To further characterize M1/M2 phenotype of RZJ/IBM cells, we will require a comprehensive analysis of gene expression in these cells upon DEX treatment.

We found that pro-IL-18, the precursor of the potent pro-inflammatory cytokine IL-18, was constitutively expressed in RZJ/IBM cells, and was slightly enhanced by the LPS stimulation. Moreover, the LPS treatment resulted in the production of mIL-18, which was released into the culture supernatant. This suggests that the nucleotide-binding domain and leucine-rich repeat containing receptor protein 3 (NLRP3) inflammasome system functions in RZJ/IBM cells, where LPS-induced activation converts procaspase-1 to its active form, caspase-1 (17). Activation of the NLRP3 inflammasome is commonly associated with the maturation and unconventional secretion of IL-1β (18, 19). However, despite the increased production of pro-IL-1β in response to LPS, we did not observe the production and release of mIL-1β under the current experimental conditions. Further investigations on the inflammasome system in RZJ/IBM cells are warranted to elucidate the reasons for this inconsistency.

Toll-like receptors (TLRs) expressed on macrophage cell membranes are well defined pattern recognition receptors responsible for pathogen recognition (20). Among them, TLR4 recognizes LPS, outer membrane components of gram-negative bacteria, and mediates LPS-induced inflammatory responses in macrophages. TLR4 expression was also detected in RZJ/IBM cells (**Supplementary Figure 1**). Regarding other TLRs, the expression levels of TLR7, TLR8, and TLR9 were obviously higher than that of TLR4 in RZJ/IBM cells (**Supplementary Figure 1**). It is interesting that TLR7 expression level was 1.9-fold higher with LPS treatment, whereas that of TLR9 was 5.1-fold lower with LPS treatment, compared to untreated cells (**Supplementary Figure 1**). Since TLR7/8 and TLR9 recognize single-stranded RNA in RNA viruses and unmethylated CpG DNA motif in DNA viruses, respectively, RZJ/IBM cells may be useful for developing *in vitro* models for virus research.

ASFV is a highly pathogenic virus against domestic pigs with a marked tropism for cells of the monocyte-macrophage lineage (21). In our recent efforts to understand the biology of ASFV and to develop a live-attenuated vaccine (LAV) against ASFV, we established three porcine macrophage cell lines of different tissue origins (7–9). Notably, IPKM cells have emerged as a useful platform for ASF vaccine development due to their ability to support virus growth (6, 22), and also for the development of a sensitive detection assay system for infectious ASFV (23). Furthermore, a naturally attenuated strain of ASFV, derived from the virulent ASFV isolate, Armenia07, through spontaneous adaptation to IPKM cell cultures, offers an alternative approach for LAV development (24). Similarly, the adaptation of a virulent virus to RZJ/IBM cells may yield unique LAV candidates for further studies. Future studies are needed to examine the potential use of RZJ/IBM cells in ASFV research.

RZJ/IBM cells showed CPE when infected with the Vero cell-adapted isolate, Lisbon60V, but not with other ASFV field isolates, including virulent strains. Despite our previous findings showing clear CPE and rosette formation in IPKM cells infected with all ASFV isolates tested in the present study (6), RZJ/IBM cells displayed less effective support for the growth of ASFV field strains, although they were susceptible to infection as evidenced by rosette formation in HAD assays. Notably, the Armenia07 isolate failed to propagate in RZJ/IBM cell cultures even at 5 dpi. The formation of a “viral factory”, characterized by virus assembly sites surrounded by vimentin cages, has been reported to play a crucial role in the propagation of ASFV (25). While the precise mechanisms underlying the suppression of ASFV replication in infected RZJ/IBM cells remain unclear, these cells have potential as a valuable tool for further investigations.

Although it has been confirmed that RZJ/IBM cells and IPKM cells are derived from macrophages, we noticed that there are differences in the effects of LPS between both cell lines. For example, LPS treatment induced a significant increase in CD169 expression in RZJ/IBM cells, but not in IPKM cells. LPS induced a production of mIL-1β in IPKM cells, but not in RZJ/IBM cells. Additionally, mRNA expression of the genes related to antiviral responses, such as IFNB1, TLR7/8, and TLR9, appears to be prominent in RZJ/IBM cells. These findings may provide insight into the ASFV replication machinery in these cell lines, since understanding the phenotypic differences in both cell lines is likely to be related to varying susceptibility to infection with ASFV isolates.

In conclusion, the RZJ/IBM cell line described herein has emerged as a practical and reliable tool for investigating the replication dynamics of ASFV and its underlying molecular mechanisms, elucidating innate immune responses in natural host species, and devising preventive strategies against devastating infectious diseases of swine, such as ASFV.

## Data Availability

The original contributions presented in the study are included in the article/**Supplementary Material**. Further inquiries can be directed to the corresponding authors.

## Supplementary material

Supplementary Figure 1 The mRNA expression of TLRs in RZJ/IBM cells. Total RNA was recovered from RZJ/IBM cells treated with or without 1 μg/mL LPS, and RNA-seq experiments were performed independently three times. The transcripts per million (TPM) values of TLR1, 2, 3, 4, 5, 6, 7, 8, and 9 genes were expressed as mean ± SEM values.
