# Supplementary figures and images for: Establishment and characterization of an immortalized red river hog blood-derived macrophage cell line
Source: Front Immunol. 2024 Sep 11;15:1465952. doi: 10.3389/fimmu.2024.1465952 (PMC11422137; doi:10.3389/fimmu.2024.1465952)

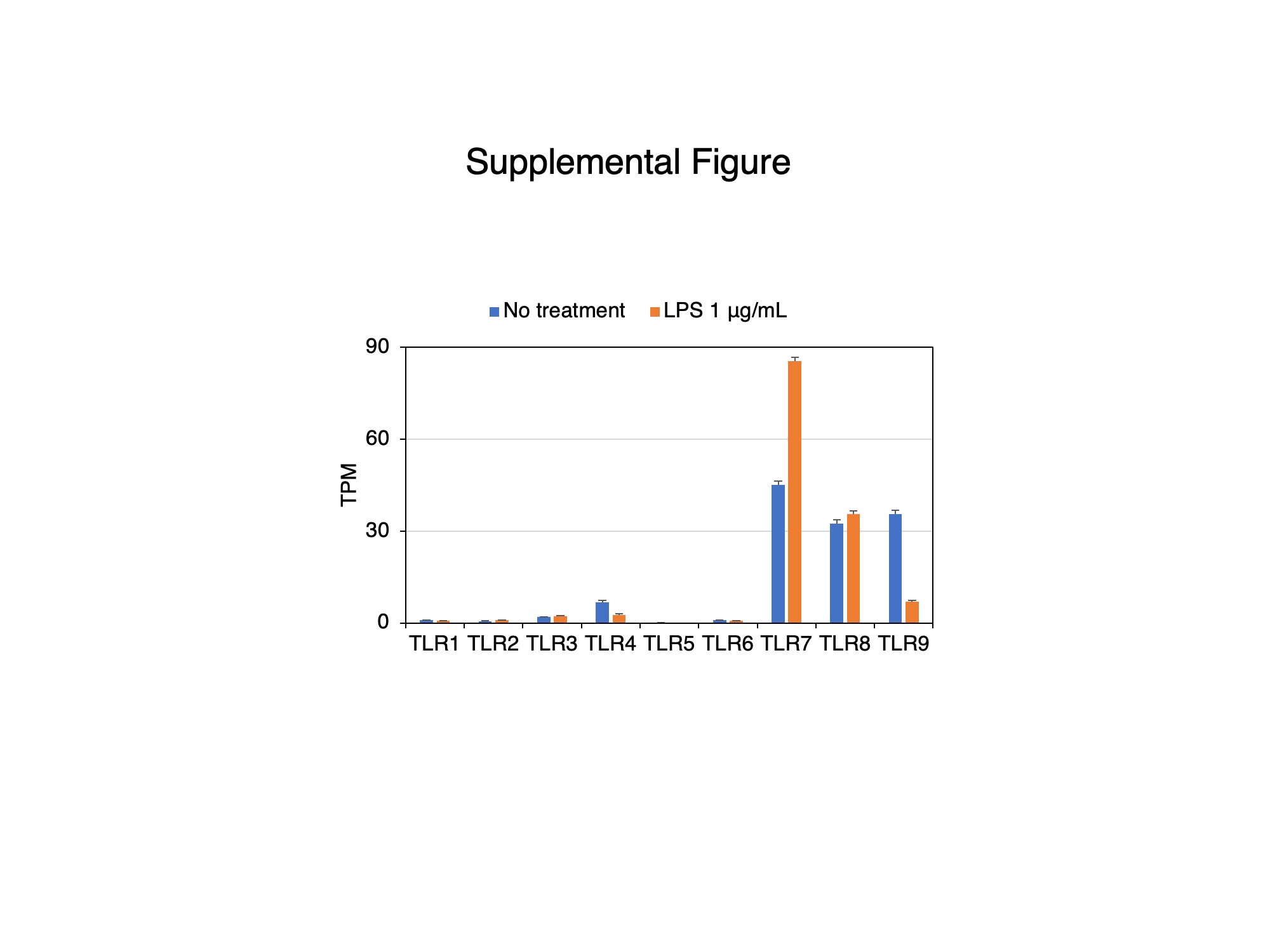

Supplement: Supplementary Figure 1 — The mRNA expression of TLRs in RZJ/IBM cells. Total RNA was recovered from RZJ/IBM cells treated with or without 1 μg/mL LPS, and RNA-seq experiments were performed independently three times. The transcripts per million (TPM) values of TLR1, 2, 3, 4, 5, 6, 7, 8, and 9 genes were expressed as mean ± SEM values. [file Image1.jpeg]
